# Iron Metabolism in the Tumor Microenvironment: Contributions of Innate Immune Cells

**DOI:** 10.3389/fimmu.2020.626812

**Published:** 2021-02-12

**Authors:** Wei Liang, Napoleone Ferrara

**Affiliations:** ^1^ Oncology, BioDuro LLC, San Diego, CA, United States; ^2^ Moores Cancer Center, University of California San Diego, La Jolla, CA, United States

**Keywords:** iron, neutrophils, macrophage, cancer, metastasis

## Abstract

Cells of the innate immune system are a major component of the tumor microenvironment. They play complex and multifaceted roles in the regulation of cancer initiation, growth, metastasis and responses to therapeutics. Innate immune cells like neutrophils and macrophages are recruited to cancerous tissues by chemotactic molecules released by cancer cells and cancer-associated stromal cells. Once they reach the tumor, they can be instructed by a network of proteins, nucleic acids and metabolites to exert protumoral or antitumoral functions. Altered iron metabolism is a feature of cancer. Epidemiological studies suggest that increased presence of iron and/or iron binding proteins is associated with increased risks of cancer development. It has been shown that iron metabolism is involved in shaping the immune landscapes in inflammatory/infectious diseases and cancer-associated inflammation. In this article, we will dissect the contribution of macrophages and neutrophils to dysregulated iron metabolism in malignant cells and its impact on cancer growth and metastasis. The mechanisms involved in regulating the actions of macrophages and neutrophils will also be discussed. Moreover, we will examine the effects of iron metabolism on the phenotypes of innate immune cells. Both iron chelating and overloading agents are being explored in cancer treatment. This review highlights alternative strategies for management of iron content in cancer cells by targeting the iron donation and modulation properties of macrophages and neutrophils in the tumor microenvironment.

## Introduction

Innate immune cells such as neutrophils and macrophages are the host’s first line of defense against invading pathogens and are responsible for initiating inflammatory responses. Recruitment and activation of adaptive immune cells to/at the infection sites are crucial steps. Cancer-associated inflammation is correlated with poor patient survival and therapeutic outcomes and is listed among the hallmarks of cancer (1). Indeed, extensive efforts have been devoted to elucidating the contribution of the innate immune system in these events. Previous studies have shown that innate immune cells, such as tumor-associated macrophages (TAM) and neutrophils, can facilitate cancer cell growth and metastasis, induce tumor angiogenesis, suppress antitumor immune response and modulate response to anticancer therapies (2, 3). These complex effects are mediated by a network of cytokines, chemokines, growth factors and enzymes released by innate immune cells that act directly on cancer cells and components of the tumor microenvironment (TME) or through cell-cell contacts between innate immune cells and cancer cells or other stromal cells (2, 3).

Recent evidence has revealed a remarkable plasticity in the metabolism of innate immune cells recruited to the TME (4). These findings perhaps are not surprising given the unique features (e.g., low pH, limited supplies of nutrients and oxygen) concomitant with aberrant accumulation of metabolism-modulating molecules in the TME. Interestingly, cancer cells can utilize metabolic byproducts from innate immune cells and other stromal cells to support cancer growth and promote drug resistance (5–8). Further understanding of how dysregulated cell metabolism in innate immune cells affects cancer behaviors is of great interest and may uncover new therapeutic avenues.

Iron is an essential element for all organisms. Its ability to be oxidized and reduced makes it ideal for transporting electrons and functioning as a co-factor in a variety of biochemical reactions in DNA synthesis (9), mitochondria respiration (10), host defenses (11) and cell signaling (12). On the other hand, this unique property may result in formation of reactive oxygen species (ROS) that have detrimental effects on genomic stability and may induce malignant transformation (13). As a result, iron metabolism is one of the key factors deciding the fates of normal and malignant cells.

This review examines the crosstalk between cancer cells and innate immune cells from the perspective of iron metabolism. We will review the evidence on how innate immune cells contribute to the dysregulated iron metabolism in cancer cells and how this process is regulated by iron metabolism and signaling molecules in the TME. We will also analyze the potential clinical benefits for therapeutic targeting the iron donation and modulation properties of macrophages and neutrophils.

## Iron Metabolism in Cancer Cells

Iron metabolism and homeostasis under physiological conditions have been reviewed in details elsewhere (12, 14). Briefly, dietary iron enters the body through absorption by divalent metal transporter 1 (DMT1) expressed on enterocytes at the duodenum of the small intestine. It can then be released to circulation through the only known iron exporter ferroportin. In the systemic circulation, the majority of the iron is bound by an iron-transporting protein named transferrin that is mainly synthesized by hepatocytes. The iron-bound transferrin (holo-transferrin) recognizes the ubiquitously expressed transferrin receptor 1 (Tfr1) or tissue-specific transferrin receptor 2 (Tfr2) and enters cells through clathrin-mediated endocytosis. Non-transferrin bound iron also exists in extracellular spaces and can be taken up by cells through transferrin receptor-independent mechanisms (e.g., DMT1). In endosomes, iron is dissociated from transferrin, reduced by six-transmembrane epithelial antigen of the prostate (STEAP) proteins and released to cytoplasm by DMT1, while transferrin receptors are mostly recycled back to the plasma membrane. Once inside the cell, iron may enter mitochondria and nucleus to participate in a series of biochemical reactions. It may also be stored in ferritin and a labile iron pool (LIP) or exported by ferroportin. Hepcidin, an iron-regulatory peptide mainly synthesized by liver, directly binds to ferroportin, resulting in the internalization and degradation of ferroportin and reduced iron export. Cellular iron homeostasis is regulated through binding of the iron regulatory proteins (IRP1 and IRP2) to the iron-responsive element (IRE) located in the untranslated region (UTR) of target mRNAs involved in iron metabolism. When intracellular iron is low, IRPs binds to IRE of Tfr1, DMT1, ferritin and ferroportin mRNAs, resulting in increased expression of Tfr1 and DMT1 and decreased expression of ferritin and ferroportin. When intracellular iron is high, IRPs is dissociated from IRE, which suppresses expression of Tfr1 and DMT1 yet permits expression of ferritin and ferroportin.

Due to increased proliferation rate and synthetic/metabolic activities commonly associated with malignancy, iron demand in cancer cells is high. To ensure ample supply, the iron uptake machinery in cancer cells is usually enhanced, while export is repressed. Transferrin synthesis acts as an autocrine mechanism supporting iron supply and growth of cancer cells (15). Overexpression of Tfr1 is frequently found in malignant tissues and is associated with worse patient survival (16–18). Inhibition of Tfr1 expression significantly blocks tumor growth and metastasis (19, 20). Moreover, upregulation of other proteins involved in iron import, such as DMT1 and duodenal cytochrome b (DCYTB), has been reported in cancer cells (21, 22). Furthermore, STEAP family members, which are highly expressed in a variety of cancer cells, can facilitate iron uptake (23–26). Conversely, expression of ferroportin is reduced in cancer cells, relative to their normal counterparts and is associated with poor patient survival (27, 28). Overexpression of ferroportin results in suppressed tumor growth (27). Elevated levels of hepcidin are found in different cancer types, further restricting ferroportin-mediated iron export and favoring iron sequestering in cancer cells (28–31). In addition, high IRP2 levels contribute to cancer cell proliferation and survival and correlate with poor patient survival (32–35).

## Iron Levels in the TME Are Regulated by Innate Immune Cells

**Figure 1:**
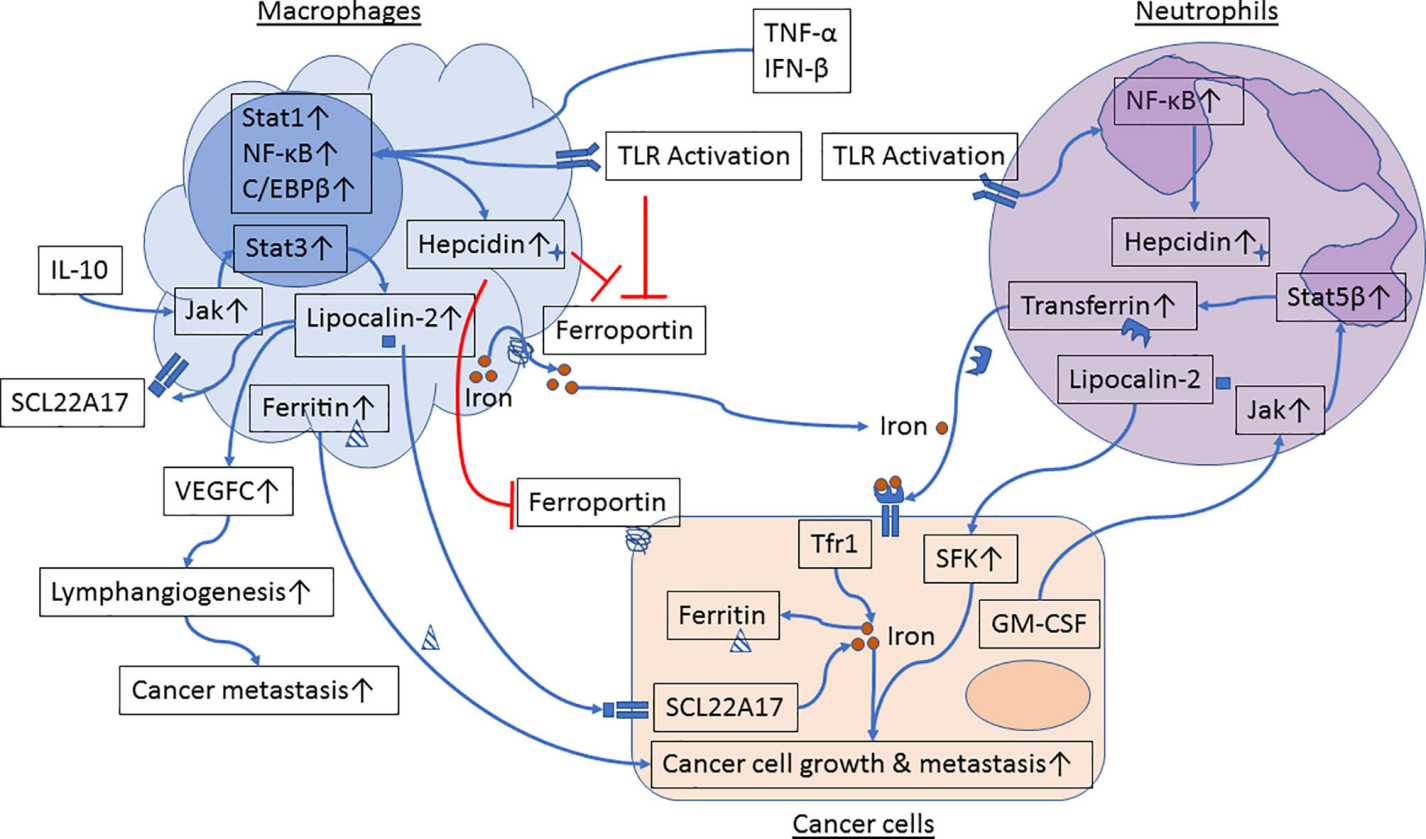
Iron metabolism in the crosstalk between cancer cells and macrophages or neutrophils. Proinflammatory cytokines and Toll-like receptor (TLR) agonists induce activation of Stat1, NF-κB and C/EBPβ in macrophages, resulting in upregulation of hepcidin and inhibition of ferroportin. In parallel, engagement of TLRs further inhibits ferroportin expression. Reduced ferroportin levels in macrophages limit iron transport from macrophages to cancer cells. IL-10 activates the Jak/Stat3 signaling pathway and upregulates expression of lipocalin-2 in macrophages. Lipocalin-2 released from macrophages can bind to its receptor in cancer cells and in macrophages to stimulate cancer cell growth and M2 polarization, respectively. Lipocalin-2 can also induce VEGFC expression, resulting in promotion of lymphangiogenesis and cancer metastasis. Moreover, macrophage-secreted ferritin directly stimulates cancer cell growth. TLR engagement induces activation of NF-κB and hepcidin expression in neutrophils. GM-CSF, produced by metastatic tumor cells, induces activation of the Jak/Stat5β signaling pathway and transferrin synthesis in neutrophils. Neutrophil-derived transferrin can promote growth of metastatic tumor cells. In addition, lipocalin-2 released by neutrophils induces activation of Src family kinases (SFK) in prostate cancer cells and enhances cancer cell migration and metastasis.

**Figure 2:**
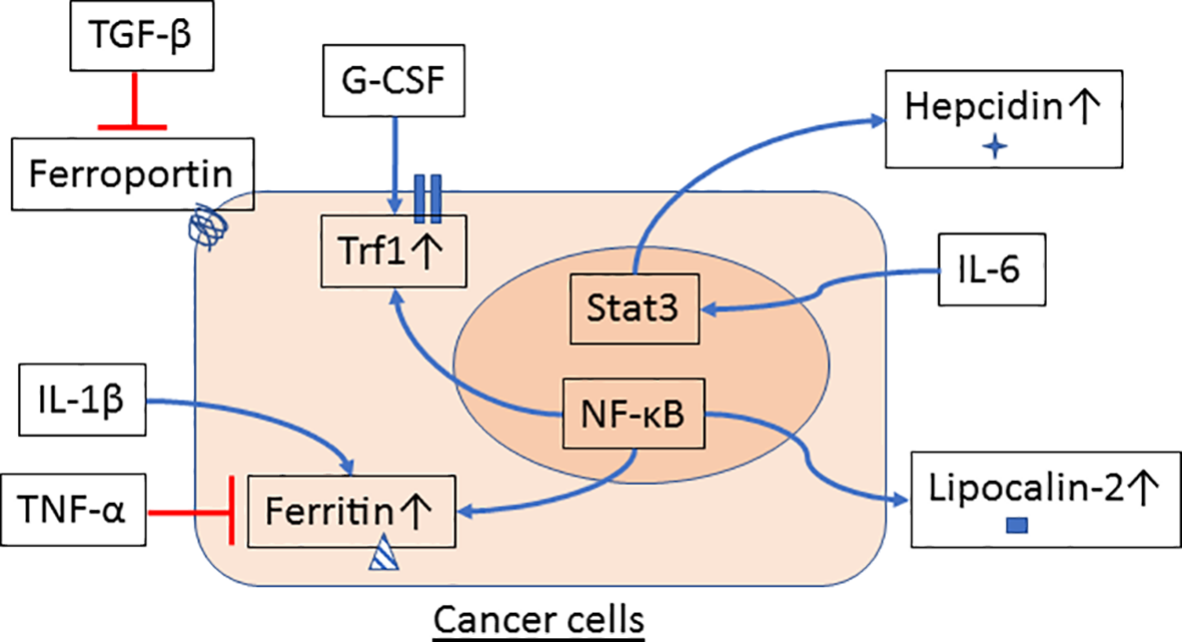
Innate immune cells signal iron metabolism in cancer cells. Cytokines and growth factors are released by macrophages and neutrophils in the tumor microenvironment (TME) and may affect the regulation of iron metabolism in cancer cells. TGF-β reduces ferroportin while G-CSF induces Tfr1 gene expression. Expression of ferritin is inhibited by TNF-α but increased by IL-1β. IL-6 stimulates Stat3 activation and hepcidin expression in hepatocytes and hepatoma cells. NF-κB, a transcription factor that can be activated by a variety of proinflammatory cytokines and factors, also participates in the regulation of gene expression of ferritin, Tfr1 and lipocalin-2 in cancer cells.

Emerging evidence suggests that innate immune cells such as macrophages and neutrophils contribute to dysregulated iron metabolism in cancer cells. Specifically, macrophages and neutrophils residing in the TME can either serve as sources of iron and iron-related proteins (**Figure 1**) or release factors that activate signaling pathways in control of iron metabolism in cancer cells (**Figure 2**).

### Macrophage and Neutrophils as Sources of Iron in the TME

Macrophages are a group of innate immune system cells with high plasticity and are frequently found in the TME. TAM can stimulate cancer cell growth, angiogenesis and metastasis, suppress anticancer immunity and render cancer resistance to therapies (36, 37). In response to different stimuli, macrophages can be polarized to M1 (classically activated, proinflammatory) or M2 (alternatively activated, anti-inflammatory) subtypes (38). Interestingly, it has been reported that M1 macrophages display high expression of ferritin and low expression of ferroportin, favoring iron sequestration in macrophages (39, 40). M2 macrophages, on the other hand, show low expression of ferritin and high expression of ferroportin, representing an iron-releasing phenotype (39, 40). Since TAM shares many features with M2 macrophages (37), it is plausible that TAM acts as a source of iron in the TME and promotes cancer growth through an iron-dependent mechanism. In fact, analysis of primary tumors and axillary lymph nodes from breast cancer patients revealed a substantial iron reservoir in stromal inflammatory cells (41). Concomitantly, while breast cancer cells display an “iron-utilization” phenotype characterized by increased expression of hepcidin and Tfr1 and decreased expression of ferritin, macrophages in primary tumors and metastasized lymph nodes manifest an “iron-donor” phenotype characterized by increased expression of ferroportin and ferritin (41). Further supporting this notion, another group examined the mitogenic effects of M1 and M2 macrophages on cancer cells and found that compared to M1 macrophages, M2 macrophage-conditioned medium has significantly greater ability to stimulate cancer cell proliferation (40). Incubation of macrophages with an iron chelator inhibits M2 macrophage conditioned medium-induced cancer cell proliferation (40). Interestingly, comparison of the mitogenic response toward M1 and M2 macrophage-conditioned media from a patient with “loss of function” ferroportin mutation showed no differences in cancer cell proliferation (40). These results suggest that the greater mitogenic activity of M2 macrophages on cancer cells is at least in part mediated by ferroportin-controlled iron release from macrophages. Additionally, M2 macrophages stimulate MCF-7 breast cancer cell growth and migration by an iron-dependent mechanism (42). Coculture with iron-loaded monocytes recapitulates the effects of iron treatment on rendering myeloma cells resistance to bortezomib (43).

Neutrophils are the most abundant immune cells in humans. Cytokines and growth factors secreted by tumor cells can stimulate neutrophil differentiation, proliferation, mobilization and release from the bone marrow (2, 44). Elevated neutrophil numbers in the systemic circulation and in tumor tissues are frequently reported in tumor patients and often correlate with poor survival (2). Like macrophages, neutrophils can be programed by tumor-released factors to exert a variety of protumoral functions by acting on cancer cells, endothelial cells, other immune cells and the extracellular matrix (2). Proteins involved in iron uptake, storage and export are expressed not only in macrophages but also in neutrophils (45). In fact, iron plays an important role in neutrophils’ defense against invading pathogens (45). Iron participates in generating the oxidative burst that is required to kill phagocytosed microbes in neutrophils (45). Moreover, neutrophils produce large amounts of lipocalin-2 and lactoferrin, both of which are iron-scavenging proteins and thus limit microbial growth (45).

Direct evidence that neutrophils are a source of iron in the TME is lacking. Yet, previous work showed that in a rat model mimicking human Alpha-1-antitrypsin (A1AT) deficiency, intratracheal administering of neutrophil elastase (NE) (a serine protease mainly synthesized by neutrophils), increases iron content in the bronchoalveolar lavage (46). NE degrades iron-containing proteins like ferritin in the extracellular space, increasing iron availability and uptake into human airway epithelial cells (46). Whether such mechanism exists in the TME and whether it contributes to increases iron uptake by tumor cells warrant further investigation.

### Macrophage and Neutrophils as Sources of Iron-Related Proteins in the TME

Lipocalin-2 is an acute phase protein that can be synthesized by a variety of cell types including epithelial cells, macrophages and neutrophils (45). Lipocalin-2 binds to bacterial or mammalian siderophores loaded with iron (47), which has two potential consequences, possibly depending on the stages of inflammation. At early stages of inflammation, binding of lipocalin-2 to the iron-siderophore complex sequesters iron from uptake by bacteria, limiting bacterial growth and thus mediating the antimicrobial function of lipocalin-2. On the other hand, the iron-siderophore-lipocalin-2 complex can serve as an iron donor and stimulate epithelial cell proliferation, an event likely occurring during the resolution phase of inflammation when epithelial cell proliferation is needed to mediate tissue repair. Given the resemblance of wound healing to cancer development, it is tempting to speculate that lipocalin-2 derived from TAM or neutrophils stimulates cancer cell growth by an iron-dependent mechanism. In fact, enhanced expression of lipocalin-2 has been documented in multiple cancer types and was associated with poor patient survival (48–52). *In vitro*, iron-loaded lipocalin-2 promotes spheroid growth of cancer cells, whereas iron-free lipocalin-2 inhibits it (48).

Macrophage-derived lipocalin-2 induces proliferation, epithelial-mesenchymal transition and metastatic potential in MCF-7 human breast cancer cells (53, 54). Besides cancer cells, lipocalin-2 can enhance the protumoral functions of tumor-associated stromal cells. Lipocalin-2, released by macrophages, induces VEGFC production, lymphangiogenesis and metastasis (55). Moreover, apoptotic tumor cells stimulate expression of lipocalin-2 in macrophages and polarization of these macrophages to M2 phenotypes (56). It remains to be determined whether the aforementioned effects are dependent on the iron donation function of lipocalin-2. In another study, lipocalin-2 was found to be predominantly expressed in TAM and to act as a paracrine factor that supplies iron, thus stimulating proliferation of cancer cells (57). Lipocalin-2 deficiency in TAM inhibits tumor growth, which can be reversed by iron supplement (58). According to a published report, iron demand increased as tumors progressed to the metastatic stage, and this was met by TAM-derived lipocalin-2 and inhibited by lipocalin-2 antibody neutralization (57). Together, these results identify TAM-derived lipocalin-2 as a promising therapeutic target for inhibiting the tumor-supporting functions of TAM. Lipocalin-2 is also secreted by neutrophils as a component of secondary granules and deficiency of lipocalin-2 impairs the chemotaxis ability of neutrophils (59). A recent report indicates that CXCL1 secreted by myofibroblasts recruits neutrophils to the TME. Neutrophil-derived lipocalin-2 induces activation of Src family kinases in prostate cancer cells and promotes cancer cell migration and metastasis (60). Whether such action is dependent on the iron-binding function of lipocalin-2 remains to be determined. Lipocalin-2 can be expressed by other cell types including cancer cells. Interestingly, a recent study reports that in the metastatic microenvironment of cerebrospinal fluid, macrophages do not express lipocalin-2 but rather produce inflammatory cytokines that induce lipocalin-2 expression in cancer cells (61). Expression of lipocalin-2 and its receptor, SCL22A17, by cancer cells was essential for cancer cell growth at metastatic sites (61).

Though typically viewed as an intracellular iron storage protein, ferritin is present in the serum. Indeed, serum ferritin has been reported to be a diagnostic and prognostic marker for inflammatory diseases and cancer (62–66). Both hepatocytes and macrophages have been implicated as sources of serum ferritin (67, 68). The releasing mechanisms and the roles of serum ferritin in inflammatory diseases and cancer are largely uncharacterized. It was shown that ferritin released by erythrophagocytosing Kupffer cells is loaded with iron and can mediate iron transport from Kupffer cells to hepatocytes (69). Moreover, in the absence of transferrin, ferritin synthesized and secreted by macrophages may serve as a source of iron for co-cultured erythroid precursors (68). These results corroborate the potential of macrophage-secreted ferritin as an iron donor for other cell types. Nonetheless, whether such hypothesis can be extended to the TME where TAM and cancer cells co-exist remains to be determined. Interestingly, TAM has been shown to be a predominant source of extracellular ferritin and TAM-secreted ferritin can act as paracrine factor that promotes cancer cell proliferation, angiogenesis and immunosuppression (70). Still, it should be pointed out that TAM-secreted ferritin can stimulate cancer cell proliferation *via* iron-independent mechanisms (71). Further studies are warranted to dissect which functions mediated by TAM-secreted ferritin are iron-dependent or independent.

Hepcidin expression in macrophages and neutrophils can be further enhanced by proinflammatory cytokines like TNF-α and IFN-β, depending on the context; or by engagement of Toll-like receptors (TLR) (72–75). Inflammation-induced hepcidin expression in macrophages is mediated by activation of the Stat1 and NF-κB pathways and induction of C/EBPβ expression (73). Secreted hepcidin can then act as an autocrine factor that inhibits ferroportin function and iron release from these innate immune cells (76). Given the abundance of endogenous TLR agonists in the TME, it will be of great interest to determine whether hepcidin released by macrophages and neutrophils in the TME, in response to endogenous TLR agonists, inhibits ferroportin on cancer cells as a paracrine factor and increases intracellular iron content and thereby cancer cell proliferation.

Transferrin and its receptor Tfr1 are the major route for cellular iron uptake. We previously reported that transferrin is expressed by neutrophils, but not cancer cells, in the metastatic microenvironment and that it mediates neutrophil-dependent mitogenic effects on cancer cells (19). Depletion of neutrophils reduced transferrin levels in the metastatic microenvironment and inhibited metastasis in mouse models (19). GM-CSF, derived mainly from metastatic tumor cells, selectively induces transferrin gene expression in neutrophils through the Jak/Stat5β pathway (19). Blockade of GM-CSF or inhibition of Jak kinases inhibits neutrophil transferrin expression and disrupted the paracrine loop between metastatic cancer cells and neutrophils, resulting in reduced metastasis (19). This work highlighted the potential of neutrophils in modulating iron metabolism in cancer cells and validated the targeting strategies for blocking prometastatic functions of neutrophils.

### Iron Metabolism-Modulating Signaling: Inputs From Macrophages and Neutrophils

Besides directly supplying iron and iron-related proteins, macrophages and neutrophils may affect iron metabolism in cancer cells through release of cytokines, chemokines and growth factors that act on cancer cells and induce changes in signaling events that regulate iron metabolism. Treatment of breast cancer cells with TGF-β, a cytokine known to be produced by TAM, reduces ferroportin expression in cancer cells (77). G-CSF, a hematopoietic growth factor that can be expressed by macrophages, induces Tfr1 expression in human myeloid leukemia cell lines (78). TNF-α can be expressed by TAM and neutrophils and treatment with TNF-α results in reduced ferritin expression in prostate cancer cells (79). TAM and neutrophils are known to produce IL-1β. Treatment of hepatoma cells with IL-1β increases ferritin expression (80). IL-6 is expressed by TAM and neutrophils and stimulates hepcidin expression in hepatocytes (81, 82). Augmented levels of hepcidin inhibits ferroportin on cancer cells and increases iron content in cancer cells (31, 83).

Numerous reports have shown that NF-κB and Stat3, two essential transcription factors regulating the inflammatory responses and immune landscape in the TME, also play key roles in controlling gene expression of iron metabolism proteins (84–88). In fact, many of the products from TAM and neutrophils in the TME are known to upregulate or downregulate activation of NF-κB and Stat3 in cancer cells (89, 90), further highlighting the crosstalk between tumor-associated innate immunity and dysregulated iron metabolism in cancer cells.

## Iron Metabolism and Macrophage Polarization

Iron metabolism is thought to contribute to macrophage polarization, although there are conflicting reports on the precise effects. For example, Agoro et al (91) reported that iron-rich diet induces M2 polarization in liver and peritoneal macrophages and suppresses the proinflammatory M1 phenotypes in mice. Addition of iron to cultured macrophages inhibited expression of M1 costimulatory proteins and prevented LPS-induced NF-κB p65 nuclear translocation and expression of iNOS, IL-1β, IL-6, IL-12 and TNFα (91). The notion that iron overload favors M2 over M1 polarization was also supported by other studies (92, 93). Yet, opposite conclusions were reached by others as it was reported that treatment of bone marrow-derived macrophages with iron induces expression of M1 markers and reduces IL-4-induced M2 markers (94). Dietary iron overload in mice leads to M1 polarization of hepatic macrophages (94). Consistent with these findings, another group reported that iron supplement promotes M1 polarization in mechanisms depending on ROS production and p53 acetylation (95). Exposure of TAM to hemolytic red blood cells or iron nanoparticles increases iron content, M1 marker expression and antitumor activities of macrophages (96, 97). Moreover, lung cancer patients with positive iron staining in tumor tissues had better overall survival associated with higher expression of markers of M1 macrophages (98). These conflicting findings highlight the complex nature of macrophage polarization even in the context of iron metabolism. They also raise some concern for the use of iron overloading or chelating strategies to switch macrophage polarization for treatment of inflammatory diseases and cancer.

## Iron Modulating Therapy: Beyond Iron Chelators

The markedly elevated demand of iron by cancer cells suggests that the use of iron chelators may be an effective anti-cancer strategy. Indeed, in preclinical models iron chelators inhibited activation of signaling pathways important for cell proliferation and survival and suppress tumor growth and metastasis (99). However, these agents demonstrated only modest therapeutic benefits when tested in cancer patients (99). While continuing efforts are ongoing to improve the bioavailability of and design combination therapies with the iron chelators, different iron-modulating strategies are warranted. The fact that innate immune cells can serve as a source of iron and iron-related proteins in the TME provided a clear rationale for target discovery and validation.

### Inhibition of Secretion of Iron and Iron-Related Proteins by Innate Immune Cells

Despite the fact that iron is critically required by cancer cells at different stages of tumorigenesis, altering iron levels and cellular uptake/storage/utilization/export machineries at systemic levels is in principle detrimental, since maintaining iron homeostasis is essential for normal physiological processes. One alternative could be to identify local sources of iron and iron-related proteins in the TME that can be pharmacologically targeted.

Our laboratory has been investigating the role of neutrophils in tumor angiogenesis and in resistance to anti-angiogenic therapies with VEGF inhibitors and these efforts led to the identification of neutrophil-derived angiogenic factors such as Bv8/PROK2 (100–102). We next sought to elucidate the nature of neutrophil-derived factors that directly promote growth of metastatic tumor cells. By employing a proteomic/functional approach, we unexpectedly identified transferrin as the major mitogen for tumor cells secreted by neutrophils (19). Depletion of neutrophils inhibited lung metastasis and transferrin production in the metastatic microenvironment. Transferrin expression by neutrophils was induced by tumor derived GM-CSF (19). The mechanism (Jak/Stat5β) by which GM-CSF induces transferrin expression is unique to neutrophils and is not shared by other cell types (19). In this case, one can expect that therapeutic agents targeting the GM-CSF/Jak/Stat5β signaling pathway (e.g., GM-CSF neutralizing antibodies or Jak kinase inhibitors) lowers transferrin levels specifically in the neutrophil-dominant metastatic microenvironment and inhibits cancer metastasis. Such strategies should spare transferrin production by other cellular sources and leave transferrin-mediated physiological iron homeostasis untouched.

Evidence reviewed above indicates that expression of lipocalin-2 in macrophages or neutrophils can promote cancer cell growth, induce M2 polarization of macrophages and is required for neutrophil chemotaxis. Therefore, blocking lipocalin-2 production from macrophages and neutrophils might simultaneously target cancer cells and cancer-associated myeloid cells. Previous work found that IL-10, an immunosuppressive cytokine that enhances the tumor-supporting functions of macrophages, induces lipocalin-2 expression in macrophages *via* activation of the Jak/Stat3 signaling pathway (53). Pharmacological inhibition of Jak or Stat3 suppresses IL-10-induced lipocalin-2 mRNA and protein levels (53). A variety of inhibitors of Jak kinases or Stat3 have entered clinical trials for cancer treatment, owing primary to the multifaceted functions of the Jak/Stat3 pathway in facilitating cancer cell proliferation, survival and metastasis, tumor angiogenesis and immunosuppression (89). It will be of great interest to determine whether such inhibitors suppress lipocalin-2 production from patient tumor-associated macrophages and neutrophils and reduce iron content in patient tumor cells and whether these effects contribute to the anticancer efficacy.

Iron exiting macrophages through ferroportin can become available for uptake by cancer cells in the TME. Reducing ferroportin expression levels in TAM thus represents a promising strategy for cancer treatment. Numerous studies have shown that stimulation of various TLRs decreases ferroportin expression, associated with increased intracellular iron concentrations in macrophages (103–105). Currently, TLR agonists are being tested in clinical trials for their ability to orchestrate anticancer immunity (106). The anticancer activities are largely attributed to TLR-mediated maturation and activation of antigen-presenting cells and follow-up activation of adaptive immune responses against cancer cells (107). It remains to be determined, in both preclinical and clinical settings, whether TLR agonists modulate the iron-donation phenotypes of TAM and reduce iron contents in cancer cells and whether such effects contribute to their anticancer activities. Moreover, TLR activation induces hepcidin expression in macrophages and neutrophils (103, 104, 108). Therefore, treatment with TLR agonist might pack a “one-two punch” by decreasing ferroportin expression and inducing hepcidin release to act as an autocrine/paracrine factor to further inhibit ferroportin-mediated iron release from macrophages and neutrophils.

### Ferroptosis-Inducing Therapy

Ferroptosis is a newly-identified, nonapoptotic form of regulated cell death, the hallmark of which is the iron‐dependent accumulation of lipid hydroperoxides (109, 110). High iron levels, inhibition of glutathione peroxidase 4 (GPX4) and glutathione synthesis, starvation of cysteine, chemotherapy and targeted therapy are known to induce ferroptosis (109, 110). It has been hypothesized that cancer cells, compared to their normal counterparts, are more susceptible to ferroptosis for the following reasons: 1) Intracellular iron levels are higher in cancer cells due to upregulated iron uptake and downregulated export mechanisms. 2) Oxidative stress is more severe in cancer cells due to activation and/or mutation of oncogenic pathways, increased metabolic activities and hypoxia in the TME. As such, therapeutic approaches that induce ferroptosis are being actively investigated for cancer treatment. A recent study, using a hyperactivated transcriptional coactivator with PDZ-binding motif-driven mouse glioblastoma model, indicates that tumor-associated neutrophils induce ferroptosis in cocultured tumor cells and necrosis in tumor tissues, leading to the enhanced cancer aggressiveness and reduced mouse survival (111). Rescue of ferroptosis through GPX4 overexpression or ACSL4 silencing in tumor cells was reported to alleviate neutrophil-induced tumor cell killing and necrosis, inhibited tumor aggressiveness and improved mouse survival (111).

One interesting question is whether cancer cell death by ferroptosis is immunogenic and whether it can modulate the phenotypes of TAM and neutrophils in the TAM. Whereas direct evidence for such a link is lacking, it was recently shown that cancer cells undergoing ferroptosis release HMGB1 (112), an endogenous TLR agonist and a known activator of immunogenic cell death (113). In fact, ferroptotic cell death after heart transplantation stimulates neutrophil recruitment through a TLR4-dependent mechanism (114).

Ferroptosis also takes place in macrophages. Numerous reports confirmed that iron overload induces macrophage ferroptosis *in vitro* and *in vivo (* 115–117). Interestingly, M1 macrophages, compared to M2 macrophages that resemble TAM, are more resistant to ferroptosis (117). Therefore, ferroptosis-inducing agents may selectively target the tumor-supporting TAM, while sparing the tumor-suppressive M1 macrophages. Inhibition of GPX4 induces ferroptosis in a variety of cell types including cancer cells (118). However, a recent study found that GPX4 deficiency does not affect survival of macrophages and neutrophils in mice (119). Instead, GPX4 deficiency induces oxidative stress and H_2_O_2_ release in myeloid cells, which promotes tumorigenesis by triggering genome-wide mutations in intestinal epithelial cells (119). This work argues against using GPX4 inhibitors, at least by itself, for inducing ferroptosis in macrophage and neutrophils. Future studies are warranted to determine the signaling pathways in regulation of ferroptosis in TAM and neutrophils in the context of cancer and whether other therapeutic approaches, alone or in combination, can eliminate TAM and neutrophils by a ferroptosis-dependent mechanism.

## Concluding Remarks and Outstanding Questions

The evidence reviewed in this article supports the notion that innate immune cells like macrophages and neutrophils promote cancer development and progression through multiple iron-dependent mechanisms. The dysregulated iron metabolism in the TME may also affect the phenotypes of innate immune cells. Though somewhat speculative, it is possible that cancer cells, equipped with hyperfunctional iron uptake and downregulated export machineries, deprive the TME of iron and produce iron metabolism-related byproducts (e.g., ROS), to boost the protumoral functions or to suppress the anticancer activities of innate immune cells.

Questions to be addressed by future studies are: what is the cause of the inconsistent results on iron-induced macrophage polarization? Given largely shared expression of iron-related proteins with macrophages, can neutrophils contribute to the release of iron and iron-related proteins and aberrantly accumulated iron levels in cancer cells, to the same extent as macrophages? Does release of iron or iron-related proteins from innate immune cells act on other tumor-associated stromal cells like T cells, fibroblasts and endothelial cells and affect their functions? What are the resistance mechanisms of innate immune cells to GPX4 inhibitor-induced ferroptosis? Does iron metabolism in TAM and neutrophils mediate response or resistance of tumor cells to chemo-, targeted- and immunotherapies; are also highly warranted.

The gained knowledge will deepen our understanding of the role of iron metabolism in the crosstalk between macrophages/neutrophils and cancer cells or other stromal cells in the TME and should facilitate the identification of novel targets for disrupting such crosstalk.

## Author Contributions

All authors listed have made a substantial, direct, and intellectual contribution to the work and approved it for publication.

## Conflict of Interest

WL was employed by the company BioDuro LLC.

The remaining author declares that the research was conducted in the absence of any commercial or financial relationships that could be construed as a potential conflict of interest.
